# Clinimetric evaluation of methods to measure muscle functioning in patients with non-specific neck pain: a systematic review

**DOI:** 10.1186/1471-2474-9-142

**Published:** 2008-10-19

**Authors:** Chantal HP de Koning, Sylvia P van den Heuvel, J Bart Staal, Bouwien CM Smits-Engelsman, Erik JM Hendriks

**Affiliations:** 1 Avans+, University for Professionals, Breda, the Netherlands; 2 Dutch Institute of Allied Health Care (NPi), Department of Research and Development, Amersfoort, the Netherlands; 3 Department of Epidemiology, Centre for Evidence Based Physiotherapy and CAPHRI Research School, Maastricht University, the Netherlands; 4 Motor Control Lab, Department of Kinesiology, K.U. Leuven, Belgium

## Abstract

**Background:**

Neck pain is a significant health problem in modern society. There is evidence to suggest that neck muscle strength is reduced in patients with neck pain. This article provides a critical analysis of the research literature on the clinimetric properties of tests to measure neck muscle strength or endurance in patients with non-specific neck pain, which can be used in daily practice.

**Methods:**

A computerised literature search was performed in the Medline, CINAHL and Embase databases from 1980 to January 2007. Two reviewers independently assessed the clinimetric properties of identified measurement methods, using a checklist of generally accepted criteria for reproducibility (inter- and intra-observer reliability and agreement), construct validity, responsiveness and feasibility.

**Results:**

The search identified a total of 16 studies. The instruments or tests included were: muscle endurance tests for short neck flexors, craniocervical flexion test with an inflatable pressure biofeedback unit, manual muscle testing of neck musculature, dynamometry and functional lifting tests (the cervical progressive iso-inertial lifting evaluation (PILE) test and the timed weighted overhead test). All the articles included report information on the reproducibility of the tests. Acceptable intra- and inter-observer reliability was demonstrated for t enduranctest for short neck flexors and the cervical PILE test. Construct validity and responsiveness have hardly been documented for tests on muscle functioning.

**Conclusion:**

The endurance test of the short neck flexors and the cervical PILE test can be regarded as appropriate instruments for measuring different aspects of neck muscle function in patients with non-specific neck pain. Common methodological flaws in the studies were their small sample size and an inappropriate description of the study design.

## Background

Neck pain is a common but significant health problem in modern society, with reported 1-year prevalence values in the world population varying from 16.7% to 75.1% for adults, with a mean of 37.2% [1]. Annual incidence rates of neck pain in general practice in the Netherlands have been estimated at 23 of every 1000 persons registered with a GP [2]. The incidence rates increase with age up to 40 to 60 years, and then decrease slightly [1,3]. Neck pain is generally more common in women than in men [1,2]. It often has a continuous or intermittent course. Approximately 30% of people with neck pain face restrictions in their activities of daily living [4]. In the Netherlands, 51% of patients with acute non-specific neck pain who consult their general practitioners are referred to musculoskeletal practitioners for treatment [5].

Panjabi et al [6] estimated that the neck musculature contributes about 80% to the mechanical stability of the cervical spine, while the osseoligamentous system contributes the remaining 20%. There is evidence to suggest that patients with neck pain have reduced maximal isometric neck strength and endurance capacity [7-10]. Furthermore, jerky and irregular cervical movements and poor position sense acuity have been found in patients with chronic neck pain [11]. Musculoskeletal practitioners apply various treatment modalities to treat patients with non-specific neck pain. Exercises are commonly used to improve neck muscle function and thereby decrease pain or other symptoms [12]. Evaluating the progress of neck muscle function during treatment requires tests which can be carried out easily and meet certain standards for clinimetric properties [13].

A 2001 review of the reliability and validity of neck muscle strength, endurance and proprioception concluded that there was a lack of reliable and valid instruments to measure strength, endurance and proprioception [14]. This review did not formulate any criteria for quality assessment, and although it included all the instruments suitable for measuring neck muscle function, it did not address cost, practicality and use of the tests. In the present review we have included only those instruments that can be easily used in daily practice (maximum of 5 minutes required for testing) and that are affordable (maximum 1000 euros). The purpose of our literature review is thus to summarise the clinimetric properties of the tests or instruments for neck muscle function in patients with neck pain which can be easily applied in daily practice.

## Methods

Studies were identified by searching the MEDLINE (through Pubmed), CINAHL and EMBASE databases for articles published between January 1, 1980 and January 1, 2007. Index terms used were: neck, cervical, reproducibility of results, reliability, reproducibility, validation studies, validity, responsiveness, muscles, isometric strength, muscle contraction, muscle endurance, muscle fatigue, dynamometry and function test.

References in retrieved documents were searched for any additional studies. The investigator (CK) screened the documents retrieved for eligibility according to the following inclusion criteria:

- The paper had to be in English or Dutch.

- Studies had to pertain to the cervical or upper thoracic spine.

- Studies had to investigate the reproducibility, validity or responsiveness of instruments or tests for measuring muscle functioning.

- The instrument or test used had to be described clearly, enabling possible replication of the test.

- The instrument or test had to be portable, affordable (maximum 1000 euros) and easy to use (maximum of 5 minutes required for testing) for healthcare professionals in daily practice.

Studies were excluded if they were non-published papers (thesis studies).

### Data abstraction and quality assessment

We investigated the following clinimetric properties: intra-observer reliability, inter-observer reliability, agreement, construct validity, responsiveness and interpretability. The data were interpreted using a checklist that was partly based on criteria developed by the Scientific Advisory Committee of the Medical Outcome Trust [15] and partly on a checklist developed by Bot et al [3] (table 1)

**Table 1:** Checklist used for the assessment of clinimetric properties of the studies included in the review

Clinimetric property	Definition	Criteria
Reproducibility	Degree to which repeated measurements in stable persons provide similar answers	K: nominal/ordinal data ICC: ordinal/parametric data
Reliability	The extent to which patients can be distinguished from each other, despite measurement error	+ Adequate design, method; intraobserver ICC > 0.85 or K > 0.41; interobserver ICC >0.70 or K>0.61 ± Information unclear or method doubtful - Adequate design, method; intraobserver ICC < 0.85 or K < 0.40; interobserver ICC < 0.70 or K < 0.60 ? No information found
Agreement	The ability to achieve the same value with repeated measurements	Limits of agreement, SEM or SDC are presented + Sufficient information, bias unlikely ± Information unclear or method doubtful - Information sufficient, instrument did not meet criteria ? No information

Construct validity	The extent to which a test actually measures the concept or trait which is being measured	Pearson's R or Spearman Rho + Adequate design, method; r >0.65 ± Information unclear or method doubtful - Information sufficient, instrument did not meet criteria ? No information

Responsiveness	Ability of an instrument to detect important change over time in the concept being measured	Hypotheses were formulated and results are in agreement + Adequate design, method; intraobserver ICC > 0.85 or K > 0.41; interobserver ICC >0.70 or K>0.61 ± Information unclear or method doubtful - Adequate design, method; intraobserver ICC < 0.85 or K < 0.40; interobserver ICC < 0.70 or K < 0.60 ? No information

Interpretability	The degree to which one can assign qualitative meaning to quantitative scores	Authors provided information on the interpretation of scores, MIC-defined Mean and SD scores before and after treatment

* K = Kappa statistics; ICC = intraclass correlation coefficient, SEM = standard error of measurement, SDC = smallest detectable change, MIC = minimal important change, and SD = standard deviation

### Description of the instruments

Descriptive data extracted from the publications included the target population and the examiners, a description of the test/instrument and the protocol used, a description of the test-retest interval, blinding of examiners for participants', each other's or reference test results, and whether withdrawals were explained.

### Reproducibility

Reproducibility is the extent to which an instrument yields stable scores over time among respondents who are assumed not to have changed [16]. Reproducibility was assessed by rating reliability and agreement. Reliability represents the extent to which individuals can be distinguished from each other, despite measurement errors. Agreement represents the absence of measurement error [16].

Weighted Kappa was considered suitable for calculating the reliability of ordinal data, and calculation of the intra-class correlation coefficient (ICC) was considered a suitable measure for ordinal or parametric data [17]. Intra-observer reliability and inter-observer reliability were rated as positive if ICC values were > 0.85 and > 0.70, respectively [13,18]. A Kappa coefficient above 0.60 for intra- and inter-observer reliability was considered positive. This is based on the Landis and Koch scale [19], which considers 0.41–0.60 to reflect moderate correlation, 0.61–0.80 substantial correlation and 0.81–1.00 almost perfect correlation. Use of the Pearson reliability coefficient was rated as doubtful, as it neglects systematic observer bias [17].

Agreement is the ability to achieve the same value with repeated measurements. In the present review, calculations of the 95% limits of agreement (LoA), standard error of measurement (SEM), smallest detectable change (SDC) or minimal detectable change (MDC) were considered sufficient. The SDC or MDC reflect the smallest within-person change in score that can be interpreted as a real change, above measurement error [3,16]. Since it is not possible to define adequate cut-off points for the result of an agreement study, a positive rating was given when an adequate method to assess agreement had been used and when authors gave convincing arguments why the agreement was acceptable [16].

### Validity

Validity is the degree to which an instrument measures what it is supposed to measure. Construct validity is the extent to which scores on a particular instrument relate to other measures in a manner that is consistent with theoretically derived hypotheses about the concept being measured [16]. Examples would be a variable, which is very similar to the variable to be validated (e.g., a muscle functioning test against dynamometry), or a variable that measures the same construct as well as other impairments (e.g., muscle functioning test against a questionnaire on self-perceived disability). A Pearson correlation coefficient or Spearman correlation coefficient above 0.65 for construct validity was rated as positive [13,18].

### Responsiveness

Responsiveness refers to the ability of an instrument to detect important change over time in the concept being measured, and is therefore considered to be a measure of longitudinal validity. There is no single agreed method of assessing or expressing an instrument's responsiveness [13,16]. Responsiveness was considered to have been adequately assessed if hypotheses had been specified and the results corresponded to these hypotheses [3]. Since it was not possible to define adequate cut-off points for the result of a responsiveness study, a positive rating was allocated when a suitable method for responsiveness had been used.

### Interpretability

Interpretability is defined as the degree to which scores and change scores can be interpreted and qualitative meaning can be assigned to quantitative scores. The articles had to provide information about the difference in scores that would be clinically meaningful. We rated this on the basis of whether the authors had presented a minimal important change (MIC) or whether information was presented that could aid in interpreting scores – for instance, presentation of means and standard deviations (SD) of patient scores before and after treatment, data on distribution of scores in relevant subgroups and relating changes in the instrument score to patients' global perceived change [3,16].

### Overall quality

To obtain an overall score for the quality of the instruments, the number of positive ratings on the above-mentioned points was summed for each instrument.

Two investigators (CK & SH) independently assessed the studies included according to the criteria list. Disagreements between the reviewers were resolved by discussion. If disagreement persisted about the assignment of a score to an item, a third person (EH) was consulted to decide on the final rating.

## Results

Searching the databases yielded 468 citation postings, of which 48 were regarded as possibly relevant and were retrieved as full articles. Sixteen studies met all eligibility criteria [20-35]. One of the reasons for exclusion was the cost of the instruments used (n = 25): various computerised dynamometers, mainly tested in university laboratories, were estimated to cost more than 1000 euros [7,8,36-58]. Studies using instruments or tests that measure propriocepsis (n = 6) [59-64] were also excluded, as were those that offered no clinimetric evaluation of the test they included (n = 1) [65] (See additional file 1).

The instruments or tests used in the included studies were: endurance tests for short neck flexors, a craniocervical flexion test, manual muscle testing of neck musculature, dynamometry and two lifting tests, viz. the cervical progressive iso-inertial lifting evaluation (PILE) test and the timed weighted overhead test. Relevant data on study population, examiners, study protocol and the results of the studies are listed in additional file 1. All articles reported on reproducibility. One article reported on the construct validity of muscle endurance of short neck flexors [30].

Disagreements between the reviewers on the quality score occurred in 22 of the 204 scores, corresponding to 89% agreement. After discussion, 3 items remained unclear and the third reviewer (EH) made the final decision.

### Muscle endurance of short neck flexors

Nine studies assessed a muscle endurance test for neck flexors with the patient in supine position. Subjects are instructed to "tuck in their chins" (craniocervical flexion) and then to raise their heads. The time between assuming the test position until the chin begins to thrust is measured in seconds with a stopwatch. This test was first described by Grimmer, and several modifications have been described since then. Three studies assessed muscle endurance of the short neck flexors as described in the first article by Grimmer, while six articles describe modifications. In these modifications, the starting position for the test is different (crook lying) and the examiners monitor the chin tuck and occipital position.

We gave the endurance test for the short neck flexors a positive rating for reliability. Eight studies used the ICC to examine reliability. Most calculated ICCs for intra-observer reliability and found them to be above the predefined value of 0.85 [21,24,25,32]. Three studies, however, reported ICCs for intra-observer reliability in healthy subjects that were below the predefined criterion (ranging from 0.76 to 0.79) [26,32,35]. The ICCs calculated for inter-observer reliability ranged from 0.57 to 1.0 [23,25,26,30,32]. One study did not use the ICC [31].

Methods used to measure agreement were SEM, LoA and MDC [23,25]. We rated agreement as doubtful. The SEM for intra-observer agreement was described in two studies involving healthy subjects, and ranged from 8.0 to 18.6 seconds (sec) [25,26]. The SEM for inter-observer agreement ranged from 2.3 to 11.5 sec for subjects with neck pain [23,25], and from 0.53 to 15.3 sec for subjects without neck pain [25,26]. Two studies reported LoA. The LoA values were -1.5 ± 6.4 sec for subjects with non-specific neck pain [23] and -2.43 and 2.33 sec in a mixed subjects group [31]. The MDC was 6.4 sec [23]. On the whole, the description of the study design and study population was acceptable. Six studies used representative examiners that had ample experience with the test [23,25,26,30,31,35], seven studies described the test-retest interval [21,23-26,30,32] and six studies gave attention to blinding aspects [21,23-26,31]. The study population in three studies consisted of subjects with neck pain [21,23,30], while three studies used mixed groups [25,31,35] and three studies included only healthy subjects [24,26,32].

Validity was analysed by comparing the results of the endurance test for short neck flexors with the Neck Disability Index (NDI) [30]. A significant association between these two measures was found by regression analysis.

### Manual muscle testing

One article described a test that is performed without head support, prone for extensors and supine for flexors. Manual resistance is applied and strength is graded 1 (i.e. enable to maintain position against gravity) to 5 (i.e. maintaining position against full manual resistance). Blizzard et al. studied the intra-observer reliability for manual testing of the long cervical flexors and extensors. In healthy subjects, the Kappa value for flexors was 0.86 and that for extensors 0.78 [21]. Because it was tested in healthy subjects, we rated manual muscle testing as doubtful in terms of reproducibility. No information was found on other clinimetric aspects of manual testing.

### Craniocervical flexion test

Upper cervical flexion, described in four articles, is measured with an inflatable pressure biofeedback unit placed behind the neck, with the patient in a supine position. The subject slowly performs an upper cervical flexion without flexion of the mid and lower cervical spine. The test can be scored in two ways. Activation score is the maximum pressure achieved and held for 10 seconds. A performance index is calculated by multiplying pressure increases from baseline (20 mm Hg) by the number of successfully completed 10-second holds. The values for the ICC measuring intra-observer reliability ranged from 0.65 to 0.93 [27-29]. Another study reported Kappa values [22]. One of the present authors (EH) recalculated this Kappa value into an ICC value of 0.84 based on the data provided in the paper. The values for the ICC measuring inter-observer reliability were 0.54 for the performance index and 0.57 for the activation score [27]. The reports on three studies which provided information on intra-observer reliability lacked essential information on the examiners, patients, the number of subjects included and blinding [22,28,29]. The study that provided information on inter-observer reliability had a satisfactory study design [27] but ICC values were below the criterion of 0.70. We therefore rated the reliability as negative. Other clinimetric properties such as agreement, validity and responsiveness were not described in the literature included in our review.

### Dynamometry

Three articles describe isometric cervical muscle strength measurements with instruments that use integrated strain gauges or a load cell and microprocessor. Results are presented in Newton. The studies we included measured neck flexion and rotation, using three different kinds of instruments [20,33,34], a Penny and Giles hand-held myometer, a portable dynamometer and a modified Sphygmomanometer dynamometer. A Pearson correlation coefficient was used for a handheld portable dynamometer [20]. The other studies presented ICCs for intra-observer reliability which were greater than 0.85 and ICCs for inter-observer reliability which were greater than 0.70 for the Penny and Giles handheld myometer and the Microfet dynamometer [33,34]. However, the study design of all three studies was incomplete. Information on blinding aspects and description of the examiner were lacking in all three articles, and only one article described the test-retest interval [33]. We therefore rated reliability as doubtful. Other clinimetric properties such as agreement, validity and responsiveness were not described in the literature we included.

### Functional lifting tests

Three articles describe two different performance tests, the PILE test [26,31] and the timed weighted overhead test [35]. In the PILE test, subjects are instructed to lift weights in a plastic box from waist to shoulder (0.76–1.37 m). After four lifting movements, the weight is increased. In the timed weighted overhead test, subjects are asked to raise their arms above their heads. They are then instructed to thread a rope with their hands through links of a chain with 5-pound cuff weights attached to each wrist. Reliability and agreement were described for the cervical PILE test and thus get a positive rating. ICC intra-observer reliability ranged from 0.88 to 0.96 and an almost perfect inter-observer reliability coefficient was reported (ICC = 1.00 (95% CI 0.99–1.0)). The intra-observer SEM ranged from 6.10 sec to 8.28 sec and the inter-observer SEM ranged from 0.77 sec to 1.19 sec, tested on three different occasions [26]. Ljungquist et al described a reliability of twice the within-subject standard deviation, with a range of 15%, as being acceptable. The percentage in the included articles ranged from 5.7% to 18.5%. The ICC for intra-observer reliability in the timed weighted overhead test ranged from 0.78 to 0.88 [35]. In general, studies focussing on function had a satisfactory design.

The rating of the clinimetric properties of the instruments included is presented in Table 2, summarising each aspect as positive, inadequate, doubtful quality or insufficient information.

**Table 2:** Summary of the quality assessment of the instruments

	Reliability	Agreement	Validity	Responsiveness	Interpretability
Muscle endurance of short neck flexors (9 articles)	+	?	?	0	0
Manual muscle examination (1 article)	?	0	0	0	0
Craniocervical flexion test (4 articles)	-	?	0	0	0
Dynamometer (3 articles)	?	0	0	0	0
Timed weighted overhead test (1 article)	?	0	0	0	0
Cervical PILE test (2 articles)	+	?	0	0	0

+ Positive

? Doubtful

- Inadequate

0 No information

## Discussion

We found eight different tests or instruments for evaluating muscle strength or endurance whose clinimetric characteristics had been evaluated. Almost all studies focussed on reproducibility, whereas one article also reported on construct validity [30]. Endurance tests for the short neck flexors were the most frequently evaluated tests. They had an acceptable reliability. The best test for the muscle endurance of the short neck flexors seems to be one in which the patient raises their head in crook-lying, while the chin tuck is monitored by the musculoskeletal practitioner. The cervical PILE test can be recommended as a functional lifting test for measuring muscle endurance, and it also has an acceptable degree of reproducibility. We do not recommend dynamometry, manual muscle examination or the time weighted overhead test, as we were rated them as doubtful.

The craniocervical flexion test [29] was developed to evaluate the muscle endurance of the deep neck flexor muscle system for its contribution to cervical segmental stabilisation, while the muscle endurance test of the cervical short muscle function was designed to evaluate the function of the superficial and deep short neck flexors. Recently, O'Leary compared isometric cranio-cervical flexion and conventional cervical flexion and found no significant differences between these two tests in the activation of the deep cervical flexion muscles. In the conventional cervical flexion test, the superficial neck flexors are dominant [66]. This means that the aims of these two tests are different. As yet, we do not recommend the craniocervical flexion test, because evidence is lacking about its clinimetric qualities. Three studies met the criteria for statistical results, but the articles lacked information on the study design as regards the description of examiners, patients, small sample sizes and blinding aspects [22,28,29]. Another study had an adequate study design, but the reliability coefficient did not meet the predefined criteria of 0.85 for intra-observer reliability and 0.70 for inter-observer reliability [27]. Overall, therefore, the results are inconsistent. Other studies related an altered electromyographic amplitude of the deep and superficial neck flexors to changes found in the craniocervical flexion test [9,67]. Although electromyography of the superficial neck muscles has been shown to be reproducible,[38,39,68] evidence for the reproducibility of measuring deep cervical flexor muscles with electromyography is lacking [67]. Therefore, the validity of the craniocervical flexion test is still doubtful, as are some other clinimetric aspects, and we can as yet not recommend using the craniocervical flexion test to measure the endurance of the short neck flexors

In contrast, the test for measuring the endurance capacity of the neck flexor muscles has, on the whole, been investigated more thoroughly and had better results for intra-observer and inter-observer reliability in particular, and can therefore be recommended.

It has recently been argued that agreement parameters, which are based on measurement error, are a purer characteristic of the reproducibility of a measurement instrument than reliability, which distinguishes between individuals and is thus more closely related to variability among such individuals. It has been postulated that agreement parameters are more suitable for instruments used for evaluative purposes, while reliability parameters are more suitable for instruments used for discriminative purposes [69]. Data on the agreement between the endurance capacity of neck flexors and the cervical PILE test have been presented in five recently published articles. The agreement scores on these tests varied. Agreement was considered acceptable when the authors gave convincing arguments for the acceptability of the agreement. This was the case in none of the included articles. Therefore, and in view of the great variation in the test scores, we rated the agreement as doubtful.

Interpretability and the responsiveness of the instruments included were not documented. Nevertheless, these items are important for evaluation purposes, because the measurement error should be smaller than the minimal amount of change considered to be important [16].

There are many types of validity. Criterion validity is accepted as being the most powerful, but in our case no gold standard was available. We therefore chose to investigate the construct validity. We found only one study that validated a modification of the muscle endurance of the short neck flexors against the NDI and found significant correlations [30]. Construct validity was rated as doubtful because of the limited number of studies and the instrument that was used, namely a questionnaire on self-perceived disability.

Some limitations of the present review should be mentioned. Firstly, some caution should be exercised when generalising the results, since only articles in Dutch or English were included. Although we did our best to track references, it is possible that we missed some studies. The reviewers were not blinded to the authors, so reviewer bias could have affected internal validity. Secondly, the criteria we used to evaluate clinimetric qualities were based on a checklist by Bot et al (2004). This list has been used previously for patient-assessed questionnaires instead of instruments to evaluate the patient's functional status [3,70,71]. This checklist was chosen for its quality and international consensus on terminology. However, compared with the original checklist, we assigned different value labels to Kappa, ICC statistics and correlation coefficients, following other authors [13,16,18,19].

The methodological quality of the design of the studies included varied. No relationship was found between the year a study was published and its methodological quality. We found both recent and older articles that provided insufficient information on methodological aspects to allow a good evaluation of the study design.

In order to ensure the external validity of a study, it is necessary to include patients with neck pain who are likely to undergo the same measurement procedure in daily practice [72]. Seven of the 16 articles we reviewed included healthy subjects [20,22,24,26,28,32,33]. Among the studies which included patients, three did not describe the inclusion or exclusion criteria [29,31,35]. Eight articles used small sample sizes (n<30) [20,22,23,26,29,31-33]. Another aspect of external validity is the inclusion of a description of the examiner and results of the examiner's training prior to the actual tests [73]. Nine articles did not mention the training or expertise of the examiner using the instrument [20,21,24,26,28,29,32,34,35]. An important aspect of internal validity is the blinding of examiners. This aspect was not well documented, especially as regards the blinding of the examiner for the status of the subject, which was only reported in four of the included studies [21,24,27,28].

A previous review applied different inclusion criteria, [14] as a result of which only four of the 16 articles included in it were re-evaluated in our systematic review. The authors included most of the studies that were excluded from our review because of the high cost of the instrument or because they measured proprioception.

The findings of our systematic review have implications for research and clinical practice. Researchers should give careful consideration to the study design and the presentation of the results. The construct validity of the muscle endurance test for short neck flexors and the cervical PILE test should be investigated by means of comparisons with other instruments that measure cervical muscle function. Future research should also report agreement parameters. Clinicians need to be aware that the endurance test for short neck flexors and the cervical PILE test should be used for different aspects of cervical muscle function.

## Conclusion

This review provides information for researchers and clinicians to facilitate choices amongst existing instruments to measure neck muscle functioning in patients with neck pain. Although the final choice of a test (or instrument) depends on the kind of muscle function to be evaluated. The muscle endurance of the short neck flexors and the cervical PILE test were found to have sufficient reliability. We therefore recommend using the muscle endurance for short neck flexors, that is patients are instructed to raise their head in a crook-lying position with monitoring of the chin tuck by the musculoskeletal practitioner, and using the cervical PILE test as a performance test.

## Competing interests

The authors declare that they have no competing interests.

## Authors' contributions

CK, SH and EH had full access to all of the data in the study and take responsibility for the integrity of the data and the accuracy of the data analysis. CK and EH: study design. CK and SH: acquisition of data. CK, SH, JBS and EH: analysis and interpretation of data. CK, SH, JBS, BS and EH: manuscript preparation. CK, JBS and EH: statistical analysis.

## Pre-publication history

The pre-publication history for this paper can be accessed here:



## Supplementary Material

Additional file 1 **Supplementary table** Click here for file
