# Supplementary material for: Clinimetric evaluation of methods to measure muscle functioning in patients with non-specific neck pain: a systematic review
Source: BMC Musculoskelet Disord. 2008 Oct 19;9:142. doi: 10.1186/1471-2474-9-142 (PMC2575213; doi:10.1186/1471-2474-9-142)
Supplement: Additional file 1 — Supplementary table [file 1471-2474-9-142-S1.pdf]

Characteristics of studies included in the review

Test	Author and Purpose	Patient	Examiner	Procedure	Results
Muscle endurance of short neck flexors	Blizzard'00 Reliability	93 subjects with neck pain and headache	Physical therapist	Patient supine, chin retracted; lift the head 2 cm. Test-retest interval: 1 month; blinded ex-pt: yes	Grimmer ICC Intra 0.96
Muscle endurance of short neck flexors	Cleland'06 Reliability Agreement	22 patients with non-specific neck pain	Physical therapist	Chin tuck neck flexion test; Test-retest interval: immediately after each other; blinded ex-pt: yes	ICC Inter 0.57 (95% CI 0.14-0.81)<br>SEM 2.3 sec. MDC 6.4 sec. LoA –1.5±6.4
Muscle endurance of short neck flexors	Grimmer'94 Reliability	93 healthy subjects	?	Patient supine, chin retracted; lift the head 2 cm; visually confirmed with vertical ruler; test-retest interval: one month; blinded ex-pt: yes	ICC Intra 0.92 (38 women), 0.93 (55 men) level association R2 0.86 women, R2 0.88 men
Muscle endurance of short neck flexors	Harris'05 Reliability Agreement	20 healthy subjects and 20 with neck pain	?	Supine hook-lying position; test-retest interval: 1 week; blinded ex-pt: no	ICC Intra healthy rater 1 0.82, rater 2 0.91<br>ICC Inter healthy test 1 0.67, test 2 0.78 subjects 0.67<br>SEM intra 8.0+11.0 sec inter no neck pain 12.6+15.3sec inter neck pain 11.5 sec
Muscle endurance of short neck flexors	Horneij'02 Reliability Agreement	22 healthy women	Physical therapist	Patient supine, hook-lying position, chin retracted; curl up head/neck 10° flexion; 2 measurements with 5-day interval (n = 22); third measurement after 16 days (n = 16); blinded ex-pt: no	Intra A1/A2 $ICC_{2.1}$ 0.79 (95%CI 0.56-0.91) SEM 15.53 sec, CV 20.1% LOA_{ratio} 0.63-1.72<br>Intra A2/A3 $ICC_{2.1}$ 0.76 (95%CI 0.45-0.91) SEM 18.61 sec, CV 23.3% LOA_{ratio} 0.56-1.77<br>Inter A1-B1 $ICC_{2.1}$ 1.0 (95%CI 1.0-1.0) SEM 0.75 sec LOA -1.52-2.70 sec<br>Inter A2/B2 $ICC_{2.1}$ 1.0 (95%CI 1.0-1.0) SEM 0.53 sec LOA -1.28-1.73<br>Inter A3/B3 $ICC_{2.1}$ 1.0 (95%CI 1.0-1.0) SEM 0.60 sec LOA 1.40-1.99s
Muscle endurance of short neck flexors	Kumbhare'05 Reliability Validity	30 subjects WAD grade II	Physical therapist & *Kinesiologist*	Patient supine in hook-lying position, head placed on 9/8 inch particle board, head in neutral position; pull board away; time in sec; test-retest interval: immediately after each other; blinded ex-pt: no	ICC inter 0.96<br>Criterion validity to NDI t=-3.1, p<0.01
Muscle endurance	Ljungquist '99	24 chronic neck and back pain	Physical therapist	Neck flexors; pt supine; Myrin goniometer fixed on the head; subject lifted head to 10˚ flexion; time (s)	Inter LOA –2.43 and 2.33<br>Intra patient 2xSD 13.4 sec (% range 22.3)

of short neck flexors	Agreement	patients and healthy controls			Intra healthy 14.6 sec (24.3)<br>Inter healthy 19.9 sec (33.2)
Muscle endurance of short neck flexors	Olson'06 Reliability Agreement	27 healthy subjects	?	Patient supine, in hook-lying position, hand under head; patient lifts head just off the fingers with chin retracted; test-retest interval: 1 or 2 days, blinded ex-pt: no	ICC intra 0.78&0.85 inter 0.83, 0.85& 0.88 (average value) SEM ranged 1.55-3.44 sec<br>Inter-subject variability: average difference between tests: 5.6-7.8 sec SD 6.2-8.4
Muscle endurance of short neck flexors	Wang'03 reliability	32 healthy subject and 30 patients with non-specific neck pain	Physical therapist	Patient supine, chin retracted; lift the head 2 cm	Short neck flexors ICC Intra healthy 0.77, neck pain 0.87
Manual muscle test	Blizzard'00 Reliability	93 subjects with neck pain and headache	Physical therapist	Long flexors/extensors, manually tested, as described by Janda; test-retest interval: 1 month; blinded ex-pt: yes	Grimmer ICC Intra 0.96<br>K Intra Flexors 0.86, extensors 0.78
Craniocervical flexion test	Chiu'05 Reliability	10 healthy subjects	?	Patient supine; inflatable air-filled pressure sensor placed behind the neck, filled to 20 mm Hg; patient slowly performs upper cervical flexion to increase pressure in 5 incremental stages of 2 mm Hg; each stage held for 10 sec; test-retest interval: 1 week; blinded ex-pt: no	K Intra 0.72, agreement 80%
Craniocervical flexion test	Hudswell'05 Reliability	40 subjects, both patients with neck pain and healthy controls<br>Intra-rater 15 subjects<br>Inter-rater 40 subjects	Osteopaths	Patient supine; inflatable air-filled pressure sensor placed behind the neck, filled to 20 mm Hg; patient slowly performs upper cervical flexion; activation score pressure achieved and held steady for 10 sec activation score, performance index amount of repetitions; test-retest interval 1 week, blinding ex-pt yes	ICC Intra Performance index 0.78 (95% CI 0.47-0.92), activation score 0.78 (95%CI 0.47-0.92)<br>ICC Inter Performance index ICC 0.54 (95%CI 0.36-0.70), activation score 0.57 (95% CI 0.37-0.72)
Craniocervical flexion test	Jull'00 Reliability	12 healthy subjects, 12 WAD > 3months symptoms	?	Patient supine; inflatable air-filled pressure sensor placed behind the neck, filled to 20 mm Hg; patient slowly performs upper cervical flexion to increase pressure in 5 incremental stages of 2 mm Hg; test-retest interval: ?; blinded ex-pt: no	ICC Intra 0.65 (22 mm Hg)- 0.89 (28 mm Hg)
Craniocervical flexion test	Jull'99 Reliability	50 healthy subjects	?	Patient supine; inflatable air-filled pressure sensor placed behind the neck, filled to 20 mm Hg; patient slowly performs upper cervical flexion; activation score pressure achieved and held steady for 10 sec activation score, performance index amount of repetitions; test-retest interval: 1 week; blinded ex-pt: yes	ICC Intra ICC 0.81 activation score, 0.93 performance index

Dynamometer	Agre '87 Reliability	8 healthy subjects	2 faculty physiatrists, 1 senior resident in physical medicine	Patient supine; neck flexion tested; break testing 3 or 4 MVC 4 sec; 1 min rest; test-retest interval:?; blinded ex-pt: no	Pearson R Intra 0.63 0.88 0.98 Inter mean 0.88 range (0.70-0.99)
Dynamometer	Philips '00 Reliability	18 healthy subjects	1 female examiner	Penny and Giles hand-held myometer; patient supine; break testing; neck flexion head raised 30˚; chin in Myometer at centre of forehead; test-retest interval: 2 weeks; blinded ex-pt: no	ICC Intra 0.965 (95%CI 0.897-0.978)
Dynamometer	Silverman'99 Reliability	30 healthy subjects, 30 with chronic mechanical neck pain	?	Microfet dynamometer, sagitally; patient supine, chin retracted, neck flexed, dynamometer at forehead, head rotated; patient supine, head rotated left and right; pain free range and chin tuck; dynamometer above ear; test-retest interval:?; blinded ex-pt: no	ICC healthy Isometric testing intra 0.78-0.89 inter 0.81-0.87<br>Break testing intra 0.74-0.89 inter 0.77-0.90<br>Wilcoxon significant difference break test neck pain compared healthy controls
Functional lifting test	Horneij'02 Reliability Agreement	22 healthy women	Physical therapist	Cervical PILE test; lift test, lift weights in plastic box from waist (0.76 m) to shoulder 1.37 m); initial 3.6 kg for women and 5.9 kg for men; 4 times, then weight increased by 2.25 kg for women and 4.5 kg for men; two measurements with 5-day interval (n = 22); third measurement after 16 days (n = 16); blinding ex-pt: no	Intra A1/A2 $ICC_{2.1}$ 0.88 (BI 0.70-0.96) SEM 8.28s CV 7.8% LOA_{ratio}0.77-1.16<br>Intra A2/A3 $ICC_{2.1}$ 0.96 (BI 0.89-0.99) SEM 6.10s CV 5.3% LOA_{ratio}0.88-1.15<br>Inter A1/B1 $ICC_{2.1}$ 1.0 (BI 0.99-1.0) SEM 1.19s LOA -3.76-3.0s<br>Inter A2/B2 $ICC_{2.1}$ 1.0 (BI 1.0-1.0) SEM 1.16s LOA -3.23-3.33s<br>Inter A3/B3 $ICC_{2.1}$ 1.0 (BI 1.0-1.0) SEM 0.77s LOA -1.80-2.55
Functional lifting test	Ljungquist '99 Agreement	24 chronic neck and back pain patients and healthy controls	Physical therapist	Cervical PILE test; lift test, lift weights in plastic box from waist (0.76m) to shoulder 1.37m); initial 3.6 kg for women and 5.9 kg for men; 4 times, then weight increased by 2.25 kg for women and 4.5 kg for men; test-retest interval: ?; blinded ex-pt: no	Inter –0.24kg LOA –2.46 and 1.82<br>Intra patient Men 2xSD 3.93kg (% range 10.5) Women 2xSD 1.19 (6.1%)<br>Intra healthy Men 2xSD 6.36kg (17%) Women 2xSD 1.71kg (8.8%)<br>Inter healthy Men 2xSD 2.12 kg (5.7%) Women 2xSD 2.12kg (10.9%)
Functional lifting test	Wang'03 Reliability	32 healthy subject and 30 patients with non-specific neck pain	Physical therapist	Timed weighted overhead test, subject stands facing work board (12 inches in height, 30 inches width), on which several chains have been strung from side to side. Subjects have to raise their arms overhead, pull a rope with their hands with 5 pound cuff weight attached to each wrist; time measured with stopwatch; test-retest interval: ?; blinded ex-pt: no	ICC Intra healthy 0.78, neck pain 0.88

EX = examiner, Pt = patient, Pearson r = Pearson correlation coefficient, Intra = intraobserver, Inter = interobserver, ICC = Intraclass correlation coefficient, MVC = maximum voluntary contraction, min = minute, SEM = standard error of measurement, MDC = minimal detectable change, LoA = limits of agreement, cervical PILE test = progressive iso-inertial lifting evaluation test, WAD = whiplash associated disorder
